# Research on soil moisture prediction model based on deep learning

**DOI:** 10.1371/journal.pone.0214508

**Published:** 2019-04-03

**Authors:** Yu Cai, Wengang Zheng, Xin Zhang, Lili Zhangzhong, Xuzhang Xue

**Affiliations:** 1 National Research Center of Intelligent Equipment for Agriculture, Beijing, China; 2 Key Laboratory for Quality Testing of Hardware and Software Products on Agricultural Information, Ministry of Agriculture, Beijing, China; 3 College of Electronic Information Engineering, Hebei University of Technology, Tianjin, China; Wroclaw University of Science and Technology, POLAND

## Abstract

Soil moisture is one of the main factors in agricultural production and hydrological cycles, and its precise prediction is important for the rational use and management of water resources. However, soil moisture involves complex structural characteristics and meteorological factors, and it is difficult to establish an ideal mathematical model for soil moisture prediction. Existing prediction models have problems such as prediction accuracy, generalization, and multi-feature processing capability, and prediction performance must improve. Based on this, taking the Beijing area as the research object, the deep learning regression network (DNNR) with big data fitting capability was proposed to construct a soil moisture prediction model. By integrating the dataset, analyzing the time series of the predictive variables, and clarifying the relationship between features and predictive variables through the Taylor diagram, selected meteorological parameters can provide effective weights for moisture prediction. Test results prove that the deep learning model is feasible and effective for soil moisture prediction. Its’ good data fitting and generalization capability can enrich the input characteristics while ensuring high accuracy in predicting the trends and values of soil moisture data and provides an effective theoretical basis for water-saving irrigation and drought control.

## 1.Introduction

Water is the primary resource that determines the survival and development of the Earth's inhabitants. Soil moisture not only plays an important role in maintaining plant growth but also is a key link in the water cycle of soil-plant-atmosphere continuum systems [1–4]. However, as human activities intensify, groundwater resources deteriorate in water quality [5,6], and the amount of excavation is significantly exceeded [7,8]. The continuous decline of groundwater levels leads to a decrease in soil water content and reduces the effective water storage capacity of the soil. Especially in dry areas, the lack of precipitation causes the soil water to not replenish in sufficient time, which negatively affects the normal growth of crops [9–11]. In this case, it is particularly important to develop an appropriate irrigation system at the right time. The growth and regression of soil moisture directly affects water consumption and growth of crops. It is an important indicator for drought resistance, flood control [12, 13], and precision irrigation decisions[14,15] in agricultural production. It is important to achieve accurate prediction of soil water regression regular patterns to properly manage agricultural water resources and promote crop yield increases.

At present, the mainstream soil moisture prediction methods mainly use empirical formulas, linear regression, and neural networks to construct prediction models. The empirical formula model is the earliest. By analyzing the initial soil water content, daily rainfall, average temperature, and daily average saturation difference, and based on the multivariate linear relationship of soil moisture, Chen Xiaofeng [16] and others established a formula for a soil moisture, precipitation, and drought assessment prediction model, which can provide drought assessment for 5 to 10 days in the future. The model provides strategies for drought-resistant irrigation systems; Jackson [17] uses the empirical formula to estimate the soil moisture flux together with a time domain reflectometry instrument (TDR). The results are similar, but the formula is simpler. Although the empirical formula is simple and easy to understand, the model parameters have strong regional dependence, and need to be recalculated when transplanting to other regions, in which is time-consuming and inefficient. With the rapid development of computer technology, various prediction models have emerged. J.W. Hummel [18] used a near-infrared reflection sensor to collect soil moisture data and analyzed the data using multiple linear regression, resulting in a predicted standard deviation of 5.31%. After the grey correlation analysis of meteorological data, Shu Sufang [19] established a linear regression model to predict soil moisture, which can show its trends. Linear regression has comparatively large errors and unsatisfactory accuracy for nonlinear data prediction owing to internal limitations and has difficulty meeting forecasting requirements. With the optimization of training algorithms, domestic and foreign scholars gradually began to use neural network algorithms for soil moisture prediction. Hou Xiaoli [20] et al. used an artificial neural network to predict soil moisture values at different depths with multi-input meteorological data, and the results were in good agreement with real data. On this basis, Ji Ronghua [21] improved the neural network activation function. The traditional activation function was replaced by a complex number domain, and the network was trained according to the multi-layer perceptron structure. The prediction accuracy improved by 9.1% compared with the traditional back-propagation (BP) neural network, providing a more accurate theoretical basis for soil moisture prediction M. Kashif Gill [22] avoided the curse of dimensionality problem in neural networks by using a support vector machine to predict soil moisture and increased accuracy to 89%. Li Ning [23] improved the neural network optimization algorithm based on the data characteristics of soil moisture. The BP algorithm has slow training speed and easily falls into local optima because the initial parameters of the network are randomly assigned. Therefore, the genetic algorithm was introduced to find the global optimal initial parameters before training, which effectively accelerate the training and improve the prediction accuracy of the model. However, soil moisture involves complex structural effects and meteorological factors, and it is difficult to establish an ideal mathematical model for soil moisture prediction. The traditional neural network’s structure characteristics and algorithms are weak for processing big data, prediction accuracy is difficult to improve further, and generalization capability and scalability are limited.

With the rapid development of artificial intelligence in recent years, in 2006, Hinton [24] proposed Deep Learning (DL), which uses a multiple hidden layer structure to increase the classification and fitting capability to big data and multi-feature data. Compared with traditional neural networks, it shows strong computing power and has been successfully applied in image recognition [25,26], search engines [27], stock price predictions [28], and other fields. Owing to the nonlinear and extremely complex nature of soil, some scholars have introduced DL into soil particle size and soil texture analysis [29, 30] in recent years, overcoming the problems of low prediction accuracy. Based on this, our aim is to construct and optimize a soil moisture prediction model through deep learning and its powerful data processing capabilities to achieve high-precision prediction of soil moisture in Beijing.

## 2. Materials and methods

### 2.1 Data acquisition and overview

The test area is located in Beijing, China (E 115°7' ~ E 117°4', N 39°4' ~N 41°6'), in Shunyi, Yanqing and Daxing. It represents a typical semi-humid continental monsoon climate in the North Temperate Zone. It is hot and rainy in summer, and is cold and dry in winter. Spring and Autumn are short. The soil texture is mainly sandy soil or resembles sandy soil. Regarding the two areas, Daxing is sandy loam, and Yanqing Shunyi is mostly medium loam. The main crops are winter wheat and summer corn. The average annual rainfall in Beijing is 585 mm, but the regional distribution is uneven, and the overall rainfall is increasing. From 2012 to 2016, the annual soil moisture change in Beijing was between 10% and 25%. The test area covers Beijing's main planting areas. The proposed model can provide a theoretical basis for water-saving irrigation strategies in Beijing.

The data used in this experiment is provided by the Beijing Meteorological Bureau and is divided into two parts: meteorological data and soil moisture data. The data includes three areas, Yanqing, Shunyi and Daxing. The period covered by the meteorological data and soil moisture data is from 2012 to 2016.The meteorological data types include daily average temperature, daily average air pressure, daily average relative humidity, daily average wind speed, daily average surface temperature, and daily precipitation; soil moisture data includes soil average mass water content at 10 cm and 20 cm depth in farmland.

### 2.2 Data processing and analysis

Different sources of meteorological data and soil moisture data result in different data formats and lengths. Data integration and matching is required. The deep learning model requires a large amount of data for training purposes and a long time-span data set to ensure complete data characteristics. The method involves selecting the training set and test set according to the amount of soil moisture data from 2012 to 2016. The integrated data contains missing values. If the missing value is included, and induces a large error, it will cause interference in the model training. Therefore, we chose to eliminate data with missing values. The final data set contains six meteorological features, as well as an initial moisture feature, and a pending prediction feature of soil moisture. After processing, a total of 1,196 data samples from Yanqing area were obtained, including 954 sets of data from 2012 to 2015 to build a training set, 242 sets of data in 2016 to build a test set, and 50 data samples were randomly selected from the test set for model selection. At the same time, a total of 239 data from Shunyi area in 2016 and 235 data from Daxing area in 2016 were used to verify the extensibility of the model.

**Fig 1:**
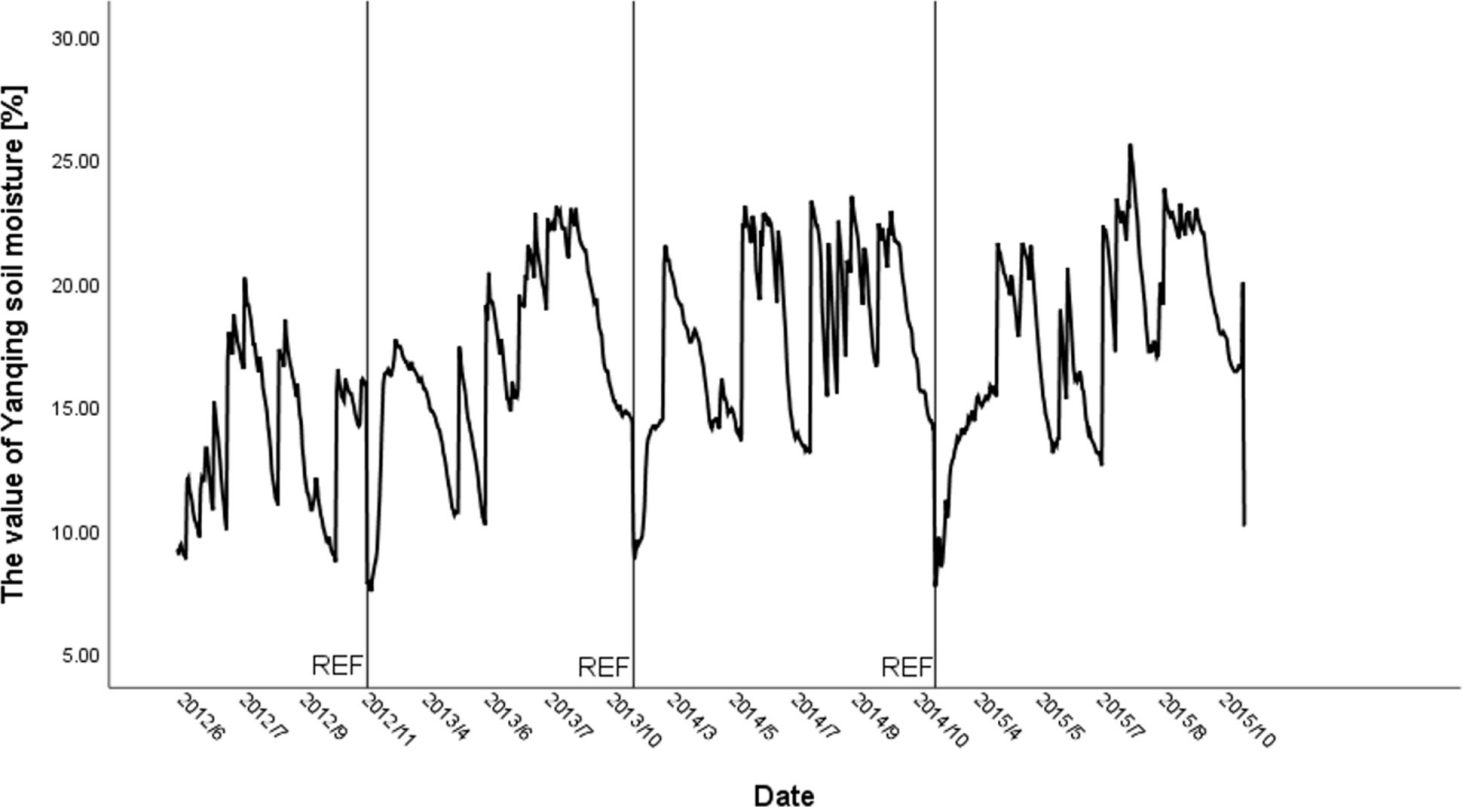
Timing diagram of soil moisture in Yanqing area.

To predict the data, we must first understand the trend of the predicted features. According to Fig 1, the water timing chart of the four years from 2012 to 2016, although the moisture data fluctuates greatly, presenting a periodical status overall, generally from July to September each year represents the data peak, the maximum soil water content is up to 25.6%. From November to February of the next year indicates the period for minimum water content, which is only 7.50%. However, different years show large discrepancies because of different meteorological conditions. Facing such complex prediction features, deep learning is suitable for soil moisture prediction because of its data fitting capabilities.

**Fig 2:**
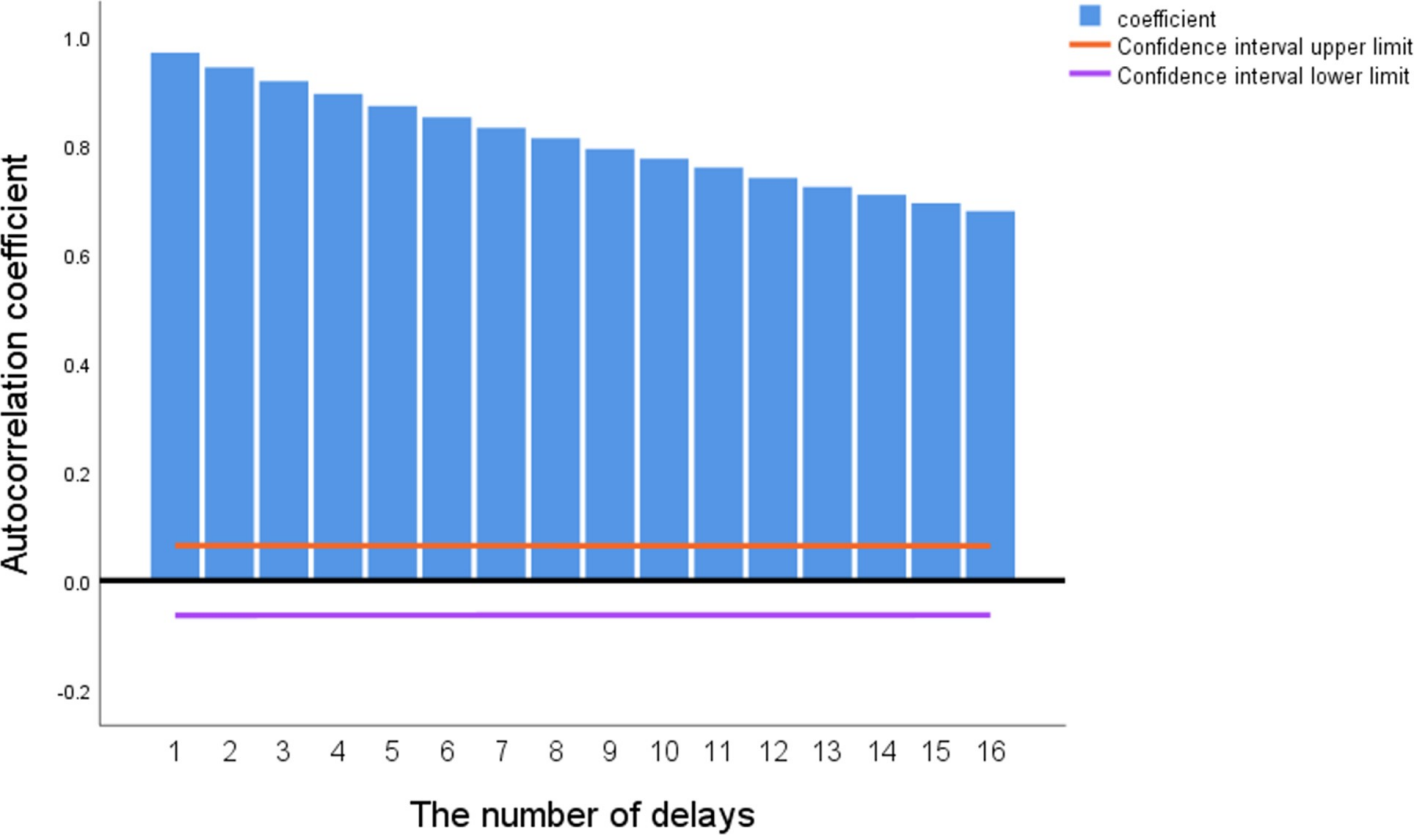
Soil moisture autocorrelation plot.

The regression prediction should be clear about the correlation between each variable and the predicted feature, so that reasonable parameter characteristics can be selected for model training. The first step is to analyze characteristics of the predicted variable. It can be seen from Fig 2 that the autocorrelation graph of the predictive feature has no rapid decay to zero with increases of the delay period, so because the soil moisture characteristic is a stationary time series. Therefore, it is possible to grasp the changing trend of soil moisture characteristics according to relevant meteorological parameters.

**Fig 3:**
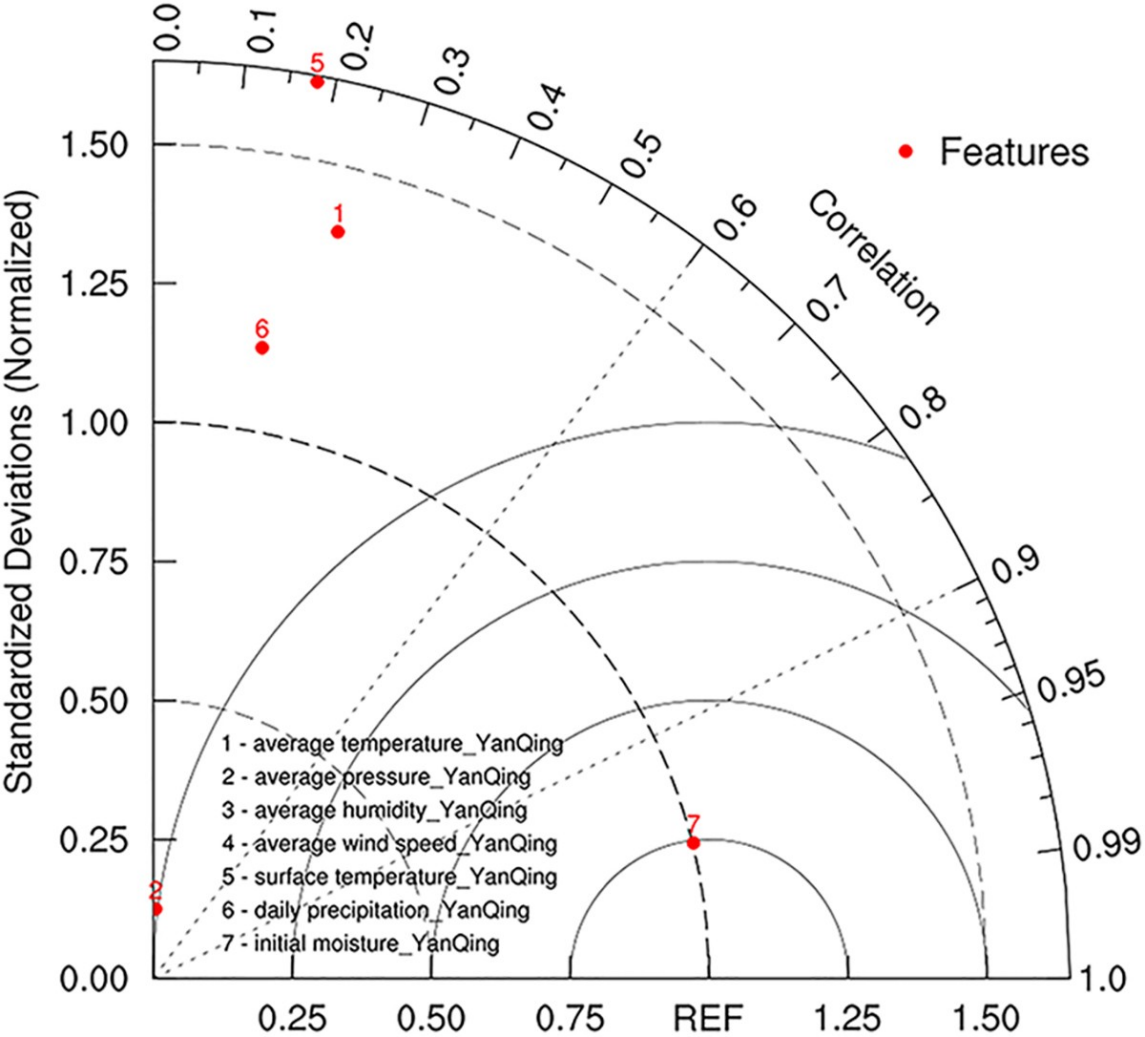
The Taylor plot of weather data.

The results of the correlation analysis between the features of the data set and soil moisture are shown in Fig 3. The reference variable of the Taylor map is the soil moisture feature (the REF point of the X-axis), and other features standard deviation divided by the standard deviation of the soil moisture are used to obtain the standard deviation ratio, which can be used to evaluate the similarity between the fluctuation range of other features and the moisture feature, and is then added into the correlation to participate in the analysis. There are seven variables to be analyzed, where points 3 and 4 (average humidity and average wind speed) are outside the standard deviation range. The data fluctuation range of these two points is more than 1.5 times the soil moisture, and exhibit data jump phenomena. Point 2 (average pressure) has a standard deviation ratio of less than 0.25 (the data fluctuation is much smaller than the moisture fluctuation range), but the correlation is the lowest. The data fluctuations of the three variables of points 1, 5, and 6 (average temperature, daily precipitation, and surface temperature) are close to the REF data. The standard deviation ratio is approximately 1.5, and the correlation is between 0.1 and 0.3. Point 7 (initial moisture) is the closest to the standard deviation ratio of the soil moisture prediction data, almost coincides with the REF line, and the correlation is close to 0.99, which indicates strong correlation characteristics. Thus, it is an essential training feature to provide maximum weight for soil moisture prediction to improve regression accuracy.

The data analysis is summarized in Table 1. It is obvious that other features and prediction variables have positive or negative correlation characteristics, which can be used to provide corresponding weights for model prediction, improve soil water prediction accuracy, and multi-feature data can be used to improve the model’s generalization capability. The above analysis indicates that the data set is reasonable for use.

**Table 1:** Correlation between various features and soil moisture.

Feature	Average temperature	average pressure	Relatively humidity	average Wind speed	Land temperature	Daily precipitation	Initial soil moisture
Soil moisture correlation	0.24	-0.03	0.39	-0.28	0.18	0.17	0.97

### 2.3 Performance evaluation measures

Four evaluation measures were selected to indicate the performance of the different models.

Mean Absolute Error(MAE) is:

$$\frac{1}{m}\sum_{i=1}^{m}\left|\left(y_{i}-{\hat{y}}_{i}\right)\right|$$ (1)

Mean Squared Error(MSE) is:

$$\frac{1}{m}\sum_{i=1}^{m}{\left(y_{i}-{\hat{y}}_{i}\right)}^{2}$$ (2)

Root Mean Squared Error(RMSE) is:

$$\sqrt{\frac{1}{m}\sum_{i=1}^{m}{\left(y_{i}-{\hat{y}}_{i}\right)}^{2}}$$ (3)

R Squared(R^2^) is:

$$R^{2}=1-\frac{\sum_{i}{\left({\hat{y}}_{i}-y_{i}\right)}^{2}}{\sum_{i}{\left(\bar{y_{i}}-y^{i}\right)}^{2}}$$ (4)

In the above formula, ${\hat{y}}_{i}$ is the predicted value, *y*_*i*_ is the true value, and $\bar{y_{i}}$ is the average value. MAE is the average of absolute errors, it can reflect the actual situation of the predicted value error. MSE is the expected value of the square of the difference between the parameter estimate and the parameter true value, it can evaluate the degree of the data change, and the smaller value of the MSE, the better accuracy of the prediction model. RMSE is the arithmetic square root of MSE. R^2^ can eliminate the influence of dimension on evaluation measure.

## 3. Model establishment

### 3.1 Model construction

**Fig 4:**
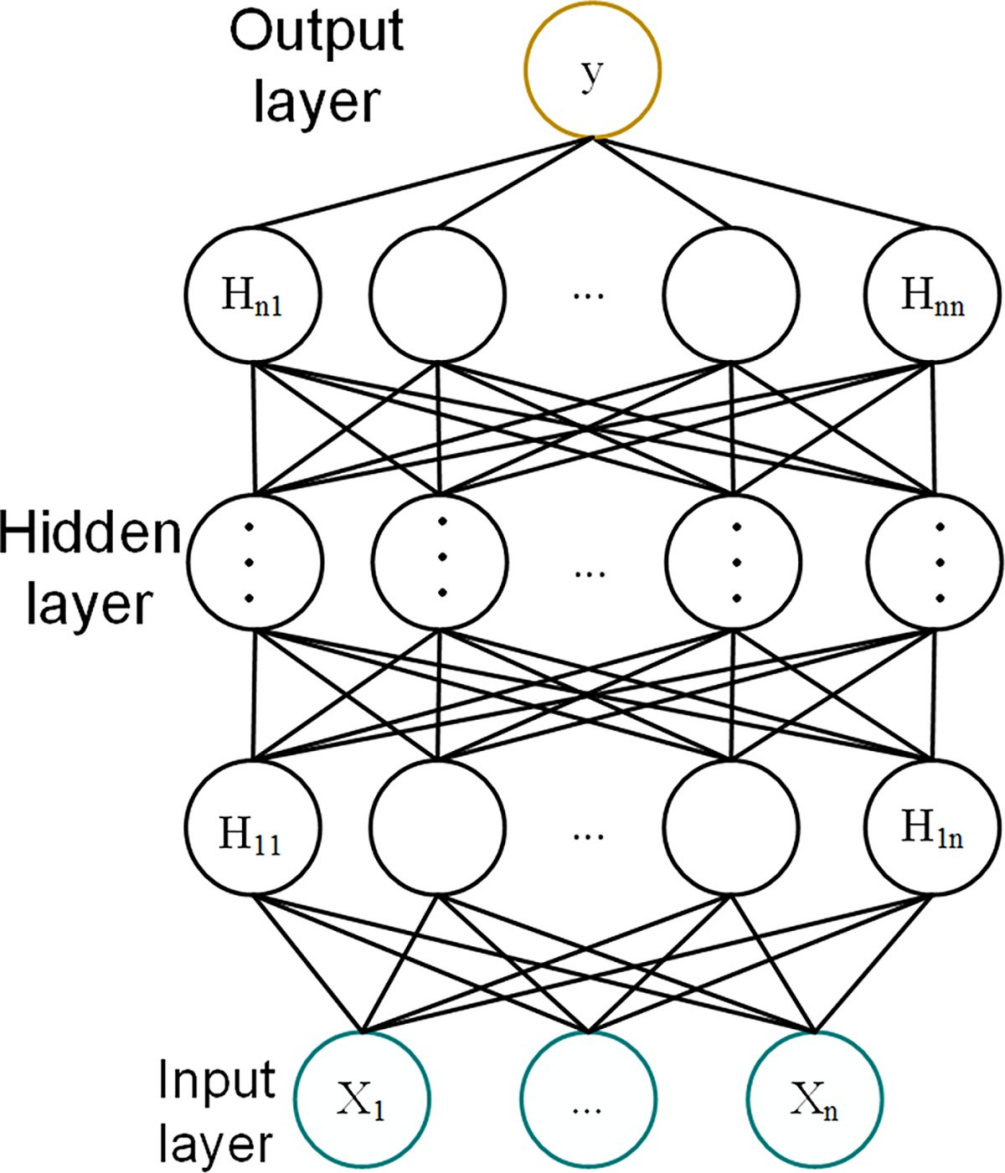
The general deep regression network structure.

Deep Neural Network Regression (DNNR) is a multi-hidden layer (at least two layers of hidden layers) regression neural network. Compared with the single hidden layer perceptron, when the same data is fitted, the increase of hidden layer depth in DNNR means the reduction of nodes in each hidden layer, which can improve data fitting capability. The advantage of the DNNR model is that it can correlate or discover feature combinations that have not appeared before, and is good at fusing hidden feature attributes, reducing the complexity of feature engineering and improving the generalization capability of the model. The DNNR network structure is shown in Fig 4.

From Fig 4, the DNNR network consists of an input layer, multiple hidden layers and an output layer. The nodes are fully connected. The number of layers can be adjusted according to the data scale. Corresponding hidden node and output layer activation functions can also be flexibly selected. The essence of the model is a combination of algorithms. The mathematical structure of the DNNR network is:

1. The number of input layer nodes is equal to the number of features of the input data. The more hidden layers, the higher the number of features needed to reduce the influence of underfitting or overfitting;
2. Each hidden layer node is composed of neurons. The neurons contain both rectifier activation and aggregation function, when constructing the DNNR model, the activation function in the default neuron is the Rectified Linear activation function, making the deep learning network neurons have sparse characteristics, which reduces the influence of overfitting while increasing the depth of the network, improving the training speed of the model, and effectively overcoming the problem of gradient disappearance. The Rectifier activation function is defined as follows:
  g(z)=max(0,z) (5)
3. The regression model output layer is different from the classification model. It is a single node. The output of the previous hidden layer is multiplied by the weight and is added to a bias on the output node to obtain the regression prediction value. The function below describes the process, where *i* is the number of nodes in the previous layer and *c* is the bias:
  $$f(x;W,c)=\sum_{i}\left(W_{i}^{T}X_{i}+c\right)$$ (6)
4. The overall function expression of the DNN model is a multi-level nested form, that is, the output of the previous layer is the input of the next layer, *x* is the input feature in the function; *w* is the weight of the layer; and *c* and *b* are node biases.
  $$f(x;W,c,w,b)=W^{T}\sum_{i}(W_{i}^{T}X_{i}+c)+b$$ (7)
5. The optimization function selected was the Adagrad algorithm. Compared to the traditional gradient descent algorithm (SGD), the same learning rate *η* is used for each training parameter. The Adagrad algorithm adaptively adjusts learning rate *η*, which must be reduced with the frequently occurring parameters to avoid parameter oscillation, and takes a larger *η* for less frequently occurring parameters to accelerate model update. It is suitable for optimizing any sparse data and perfectly matches the characteristics of the above Rectified Linear activation function. ∇_*i*,*t*_*J*(*θ*) is the gradient of the i-th parameter in the t-th round; *ε* is the minimum value; G_*i*,*t*_ is the accumulation of the previous t-step *θ*_*i*_ gradient; The expression is as follows:
  $$\theta_{i,t+1}=\theta_{i,t}-\frac{\eta }{\sqrt{G_{i,t}+\varepsilon }}\nabla_{i,t}J(\theta )$$ (8)

### 3.2 DNNR model training and optimization

The DNNR model training involves supervised training, in that the training set and the test set features all need labels, and the model parameters (weights and biases) are adjusted according to the comparison between the model prediction results and the labels to minimize the error. Training is stopped when the maximum number of specified training steps is reached or the preset accuracy is met.

The number of hidden layers and the number of hidden layer nodes can directly affect the training speed and prediction accuracy of the model. This paper uses six meteorological data features and one soil water content feature to predict soil moisture. So the number of input layer nodes is 7, which is equal to the number of features; the output layer sets the number of nodes (according to the regression characteristics) to 1; and because the data size is medium, two hidden layers in the hidden layer structure are sufficient to meet the requirements. The numbers of first layer and second layer hidden nodes need to be evaluated and selected through multiple rounds of testing. The comparison results are shown in Table 2.

**Table 2:** Comparison of training results of different model structures.

Model construction	Train steps	Train loss	Test Loss	Average train loss	Average test loss
7-50-100-1	15000	0.99	0.58	0.92	0.96
		0.85	1.31		
		0.91	1.00		
7-50-50-1	15000	0.95	0.55	0.92	0.81
		0.94	0.88		
		0.87	1.08		
7-100-50-1	15000	0.63	0.46	0.63	0.68
		0.66	0.73		
		0.61	0.86		
7-50-25-1	15000	0.84	1.29	0.86	1.31
		0.77	1.44		
		0.97	1.21		
7-150-100-1	15000	0.51	2.24	0.56	1.42
		0.58	1.08		
		0.60	0.94		

**Fig 5:**
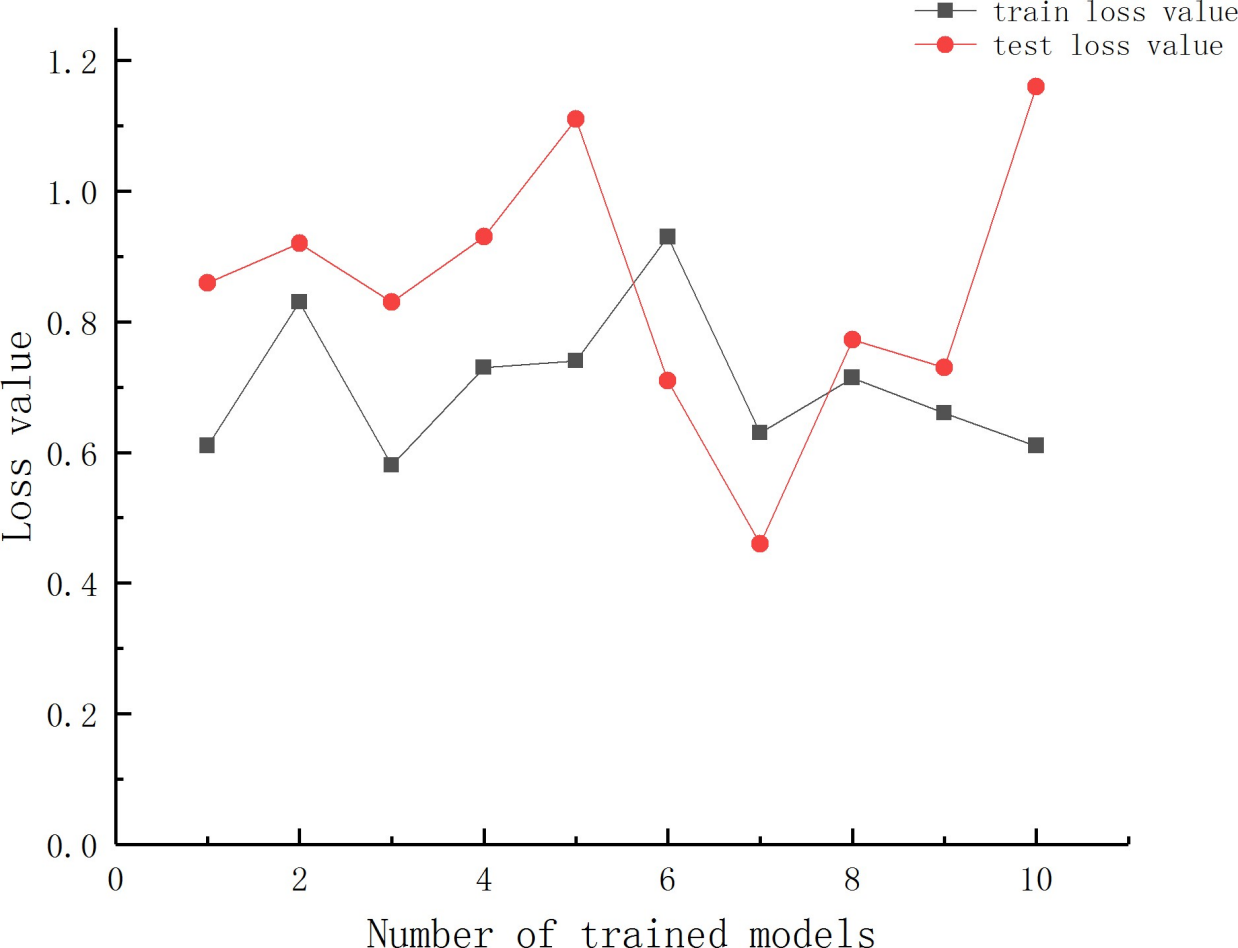
The DNNR model training results(ten times).

It can be seen from Table 2 that each model structure is trained three times, in the comparison of the number of hidden layer nodes, the first layer nodes are connected with the input layer and is responsible for learning the characteristics of the data set, the second layer nodes are responsible for fitting the learned characteristics, so if the number of nodes is much larger than the number of features it will cause information redundancy. Conversely, fewer nodes can cause under-fitting. This affects the training accuracy of the model. The above theory is consistent with the results shown in Table 2. Therefore, the number of nodes in the first layer of the model is selected as 100. The second layer is selected as 50. Based on the above analysis, a 7-100-50-1 model was finally selected. After determining the model structure, ten models training operations were repeated to select the best results in multiple experiments. The results are shown in Fig 5.

As can be seen from Fig 5, since the model weights are initialized with a random process, the results of the ten models training are different, and the training loss value and the test loss value fluctuate within a range of [0.4, 1.2], so the lowest model training loss value as the selection, which training loss value is 0.63 and the test loss value is 0.46.

In order to prove the performance of the selected model, the sliding window with data length of 50 is set, the moving step is set to 10, in the case where the window slides to the end of the data and the amount of data is less than 50, the amount of missing data is complemented from the beginning of the test set. the method can select 25 sets of test data from the test set with the data length of 242, and the test data volume of each set is 50, and the performance is verified by inputting the model separately. The test results obtained 25 test loss values. The single sample t-student test was used to analyze the 25 test loss values and the training loss values. Under the premise of 95% confidence interval, the obtained bilateral Sig value was 0.51>0.05. At a significant level of 0.05, there was no significant difference between the test loss value and the training loss value, indicating that the trained model has good generalization ability. The specific analysis results are shown in Table 3.

**Table 3:** Single sample t-student test.

	t	Degree of freedom	Average difference	Sig.
Test data	-0.669	24	-0.039	0.510

## 4. Results

**Fig 6:**
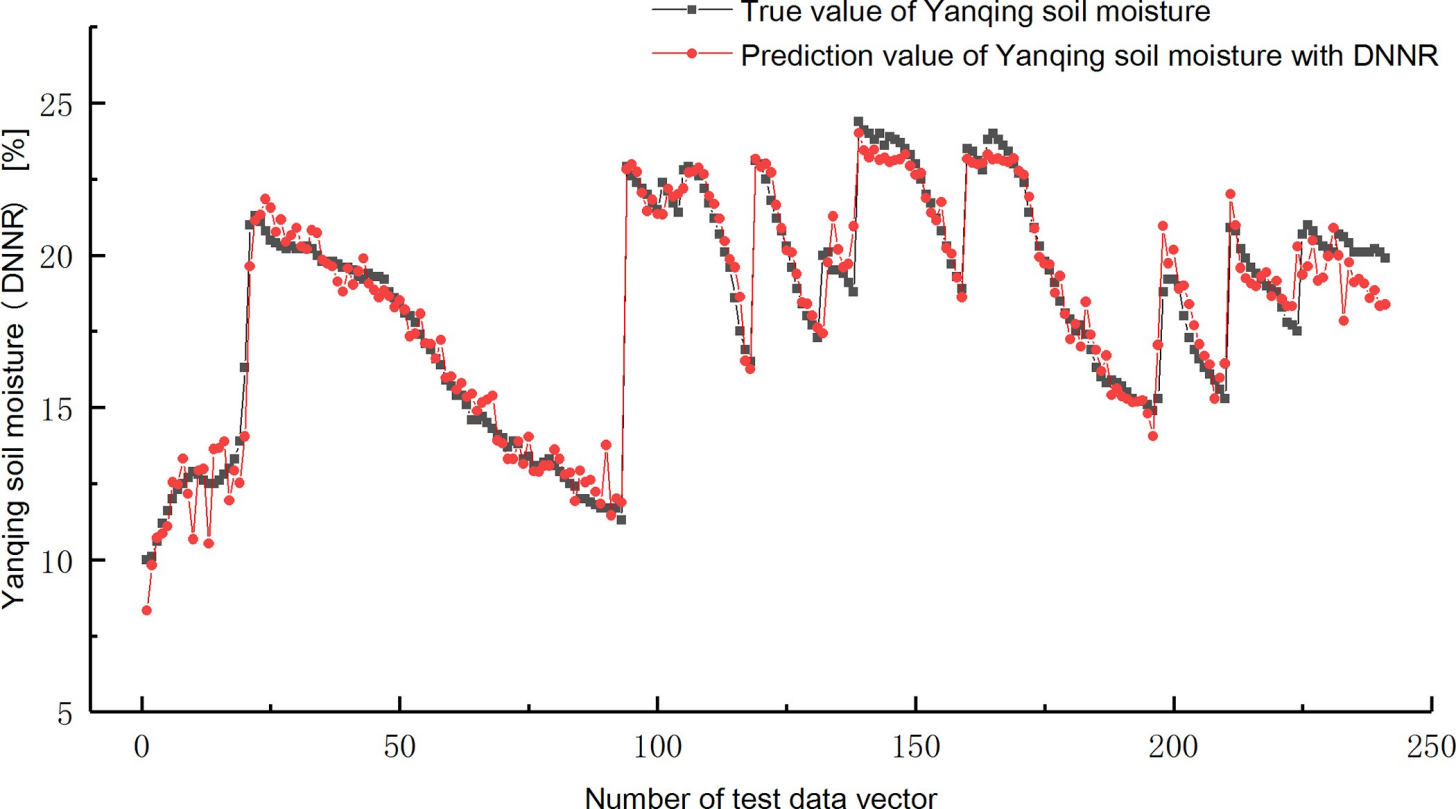
The comparison of daily soil moisture prediction in Yanqing (DNNR).

To verify the generalization capability of the constructed model, all the 242 sets of data in the test set were selected for prediction experiments. The prediction results are shown in Fig 6. The soil moisture prediction value is consistent with the true value, and 92.56% of the data prediction error is within ±1. The predicted value is higher than the true value. The prediction of soil high water content data (data points with water content of 15% or more) is accurate, where the minimum relative error is 0.06% and the maximum is only 8.75%. The prediction of low water content data (data points with water content of 15% or less) exhibits somewhat higher prediction error, where the maximum relative error is 17.29% and the minimum is 0.58%. It remains within a stable acceptable error range, and the average relative error is 0.57, which ensures that the soil moisture data predicted by the model can be used in actual guidance in Yanqing.

**Fig 7:**
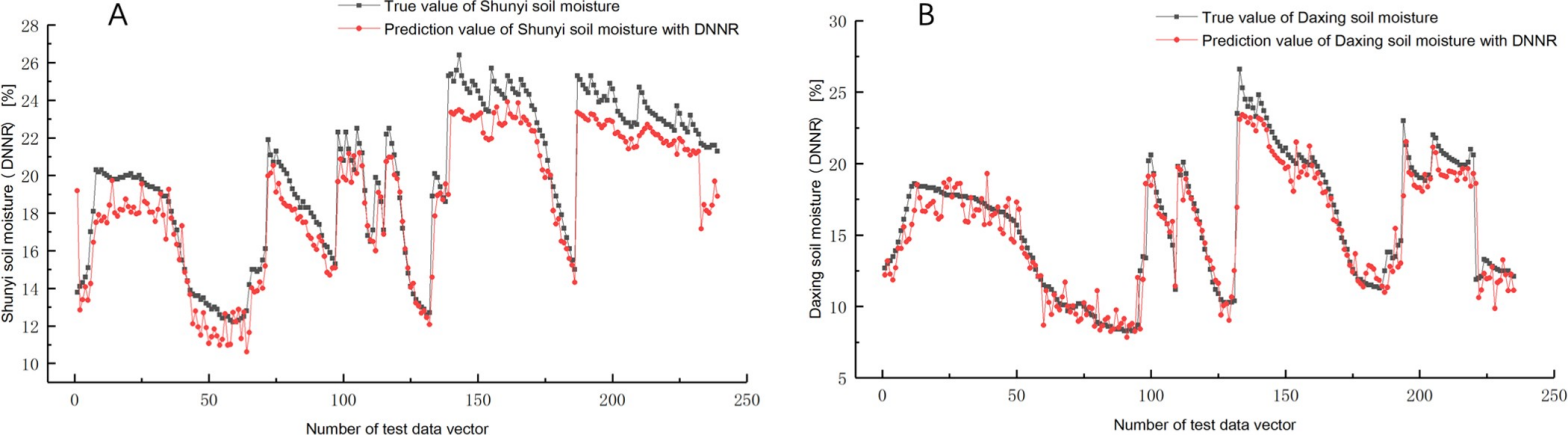
The comparison of daily soil moisture prediction in Shunyi and Daxing (DNNR). (A)Comparison of daily soil moisture prediction in Shunyi.(B)Comparison of daily soil moisture prediction in Daxing.

At the same time, the model is also used to predict soil moisture data in the Daxing and Shunyi areas. The previously constructed Shunyi area test set (a total of 239 sets of data) and the Daxing area test set (a total of 235 sets of data) were input into the prediction model for model scalability verification. The prediction results are shown in Fig 7. The true value range of soil moisture in Shunyi area from 12.2 to 26.4, and the range of prediction value from 10.6 to 23.9. The true value range of soil moisture in Daxing area from 8.3 to 26.6, and the range of prediction value from 7.8 to 23.4. It can be seen that the extreme value of prediction in other areas to the soil moisture are lower than the true value.

DNNR model error analysis results are in Table 4. The average absolute error of Shunyi prediction is 1.33, and the overall prediction value is lower than the actual value. However, the predicted value and the true value have a strong Pearson correlation of 0.97. The average absolute error of Daxing prediction is 1.03, the overall predicted value is close to the true value, and the predicted value and the true value have a strong Pearson correlation characteristic of 0.96.

**Table 4:** Multi-region prediction error analysis of DNNR model.

AREA	Evaluation Measures	DNNR
Yanqing	MAE	0.57
	MSE	0.61
	RMSE	0.78
	R^2^	0.98
Shunyi	MAE	1.33
	MSE	2.58
	RMSE	1.61
	R^2^	0.97
Daxing	MAE	1.03
	MSE	1.97
	RMSE	1.40
	R^2^	0.96

**Fig 8:**
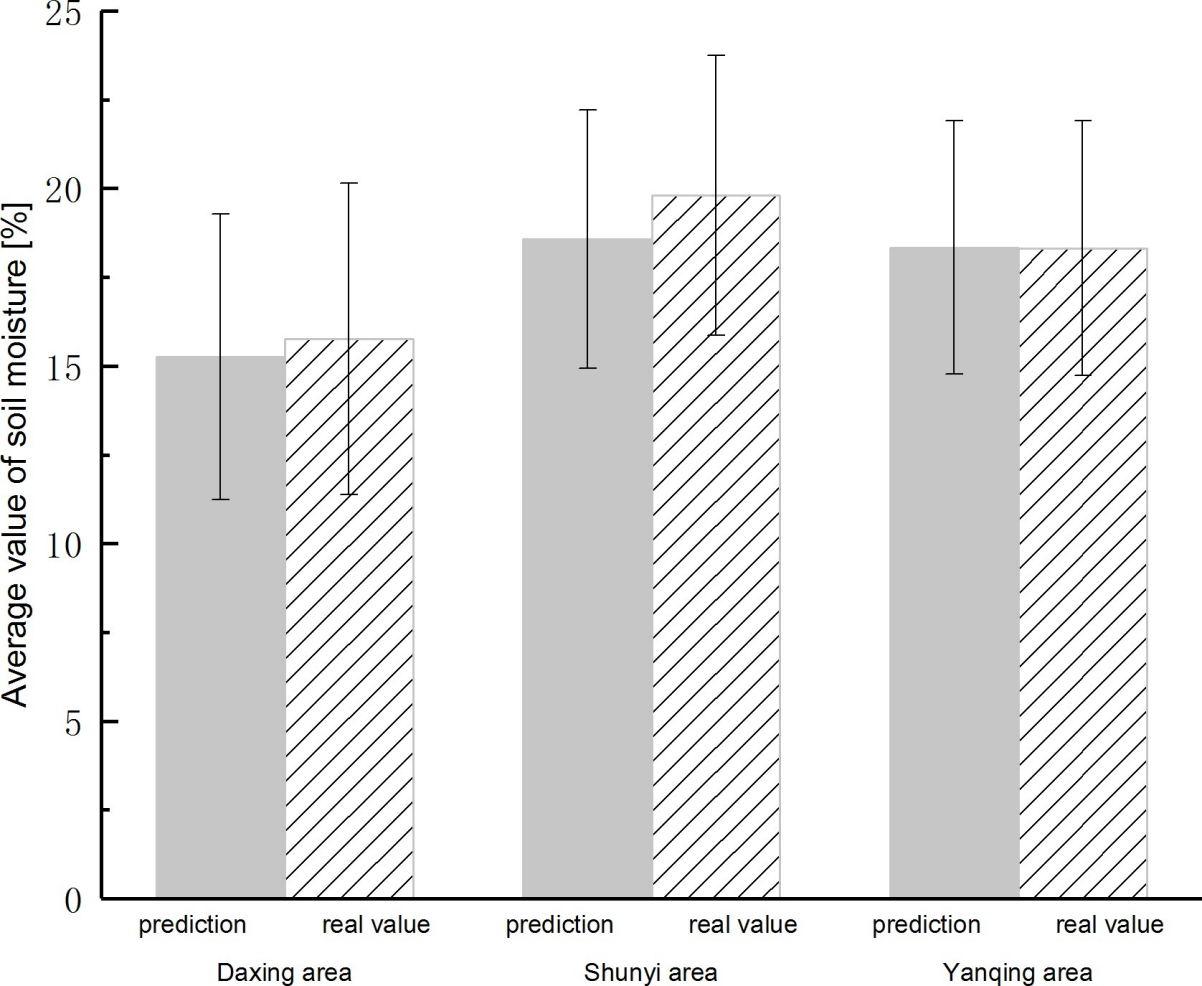
The comparison for average soil moisture predictions and real values in three regions(DNNR).

The above analysis can clearly see that because the soil moisture has regional characteristics, the predicted values of other regions contain different degrees of error, the further statistical analysis of the prediction for the three regions are shown in Fig 8. The average value of the raw soil moisture of Yanqing area is 18.32%, the average value of prediction is 18.34%. The average difference is only 0.02%, It indicates that the soil moisture value can be accurately predicted and has very close data center trend. The average value of the raw soil moisture of Shunyi area is 19.80%, the average value of prediction is 18.58%. The average and predicted values of soil moisture in this region differ by more than 1%, but it is still acceptable. The average value of the raw soil moisture of Daxing area is 15.77%, the average value of prediction is 15.26%. The average difference is weaker than the Yanqing area but better than the Shunyi area. It can also accurately predict the soil moisture data values.

The above results indicate that the model has great generalization capability and remain within a stable acceptable error range, which ensure that the soil moisture data predicted by the model can be used in actual guidance in Beijing.

## 5. Discussion

The location of this test was in Beijing, because soil water movement is a complex time series system, and its changes are closely related to regional climatic conditions and ecological environments, with obvious random fluctuations, and the differences of soil moisture regression regular patterns have large divergence. Therefore the discussion in this paper is mainly focused on a domestic soil moisture model evaluation. The input variables of the existing soil moisture prediction model are selected from air temperature, air humidity, atmospheric pressure, soil moisture, daily precipitation, illumination duration, radiation intensity, average wind speed, and initial soil moisture [16–22]. The different model characteristics require different input variables, so proper selection of variables (among those above) is also one of the keys for accurate soil moisture prediction [7,10,18]. Selecting appropriate meteorological parameters as the input features of the model can significantly improve the accuracy of soil moisture prediction. With the rapid development of the agricultural Internet of Things, the types and quantities of monitoring data are constantly increasing. Thus, a model must have sufficient data compatibility and expandability while ensuring the accuracy of prediction. At the same time, soil moisture has strong regional characteristics, which make it difficult to directly compare the performance between prediction models constructed using different regions and their corresponding datasets. It is necessary to use the evaluation indicators as qualitative and quantitative measurement criteria to analyze the advantages and disadvantages of different models. Therefore, the selection of input features and models, and the evaluation of model performance after being fully constructed are issues that need to be addressed,

Using SPSS to analyze the autocorrelations of moisture data found that it is a non-stationary time series, indicating that the water content is affected by other meteorological parameters. Increases in air/soil temperature, light, and wind speed will accelerate the evaporation of soil surface water, which is a negative correlation parameter. Soil/air humidity, atmospheric pressure, and rainfall increase soil moisture, which is a positive correlation parameter. The rainfall factor has the most direct impact and greater amounts of rainfall can directly saturate the soil moisture. Existing models all select the initial moisture as the input feature, and other input feature selections will have larger differences. Ji Ronghua [20] and others analyzed the rainfall, temperature, and wind speed in the western part of Cangzhou City, Hebei Province, and only selected the most relevant rainfall data. The correlation coefficient (R^2^) was 0.88, so the prediction model input only contained rainfall and initial moisture. After we analyzed the soil moisture data in Yanqing, Beijing, the correlation between rainfall and prediction characteristics is 0.17, and the standard deviation ratio is 1.5, indicating that the influence of meteorological parameters in different regions is significantly different. Hou Xiaoli [19] and other researchers selected five features: temperature, wind speed, duration of sunshine, humidity, and precipitation as input. The correlation of the soil moisture content at 20 cm depth was predicted by a multi-layer perceptron (MLP) model to be 0.98, which is same as the correlation prediction in this paper of 0.98, although the dataset is different. The DNNR model we used has seven input features, indicating that the DNNR model can maintain prediction accuracy while enriching the feature types. Shu Sufang [18] et al. defined 17 meteorological factors to analyze the correlation with soil moisture in the Jinhua area. Finally, 5 mm precipitation and evaporation differences were used to construct a linear regression model to predict soil relative humidity. The average relative error at 20 cm depth prediction was 6.89%, which was higher than the 0.57% of the DNNR model. It can be seen from the above analysis that a reasonable increase of input parameters can improve the prediction accuracy of the model, and the prediction accuracy of multivariate data is higher using variables that are easy to obtain from conventional soil moisture monitoring stations.

To verify the superior performance of the DNNR model, we compared it with existing models, most of the soil moisture prediction models are LR(Linear Regression), SVM(Support Vector Machine), ANN(Artificial Neural Network) and related improvement models, the R^2^ of DNNR model is higher than SVM and ANN1 by 9% and 24%, the RMSE of DNNR model is less than SVM and ANN1 by 80.74% and 87.02%, the MAE of DNNR model is less than LR, SVM, ANN1 and AGNN by 91.73%, 84.38%, 88.51% and 54.76%, the comparison results are shown in Table 5, that the DNNR model constructed in this paper is superior to the above model in the evaluation of comparison with multiple performance measures.

**Table 5:** The comparison of multi-model prediction evaluation measures.

Model	R^2^	RMSE	MAE
LR[19]			6.89
SVM[22]	0.89	4.05	3.65
ANN1[22]	0.74	6.01	4.96
ANN2[20]	0.98		
AGNN[23]			1.26
DNNR	0.98	0.78	0.57

Although the model has certain advantages in specific measures, the conditions are different for each model, and the composition of the data set and regional differences are difficult to eliminate. To solve this problem, this paper constructs a neural network model using the same data set used for the DNNR for comparison purposes. MLP is one of the most widely used advanced models, and it is more convincing to choose this model for comparison.

An MLP model was constructed using six meteorological features and an initial moisture feature. The MLP model is a 1-100-1 three-layer network consisting of a single hidden layer. The activation function of the hidden layer node is a hyperbolic tangent (Tanh). The training features types and quantities are the same as those in Table 1.

**Fig 9:**
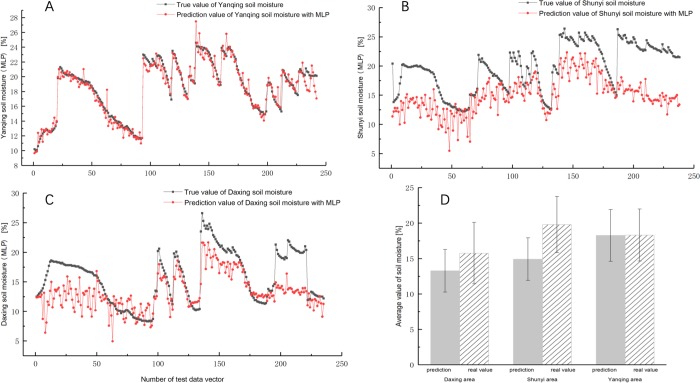
The comparison of daily soil moisture predictions in Yanqing, Shunyi and Daxing(MLP). (A) Comparison of daily soil moisture prediction in Yanqing.(B) Comparison of daily soil moisture prediction in Shunyi. (C) Comparison of daily soil moisture prediction in Daxing.(D) The comparison for average soil moisture predictions and real values in three regions.

First of all, the soil moisture in Yanqing area, Shunyi area and Daxing area of Beijing was predicted and shown in Fig 9. It can be seen from Fig 9 that the MLP model predicts the soil moisture in the Yanqing area with a correlation coefficient of 0.97, which is only lower than the 0.98 of the DNNR model, but the prediction errors of the other two regions are larger. The predicted values of Shunyi area and Daxing area are significantly lower than the raw soil moisture data, and the correlation coefficients are 0.70 and 0.75, respectively, which is much lower than 0.97 and 0.96 of the DNNR model. In Fig 9D, the average value of the raw soil moisture of Yanqing area is 18.32%, the average value of prediction is 18.27%. The prediction effect of Yanqing area is similar to the DNNR model. But the average value of the raw soil moisture of Shunyi area is 19.80%, the average value of prediction is 14.92%. The average value of the raw soil moisture of Daxing area is 15.77%, the average value of prediction is 13.28%. The average errors of the predictions in the other two regions accounted for 24.65% and 15.79% of the raw soil moisture data, respectively.

**Fig 10:**
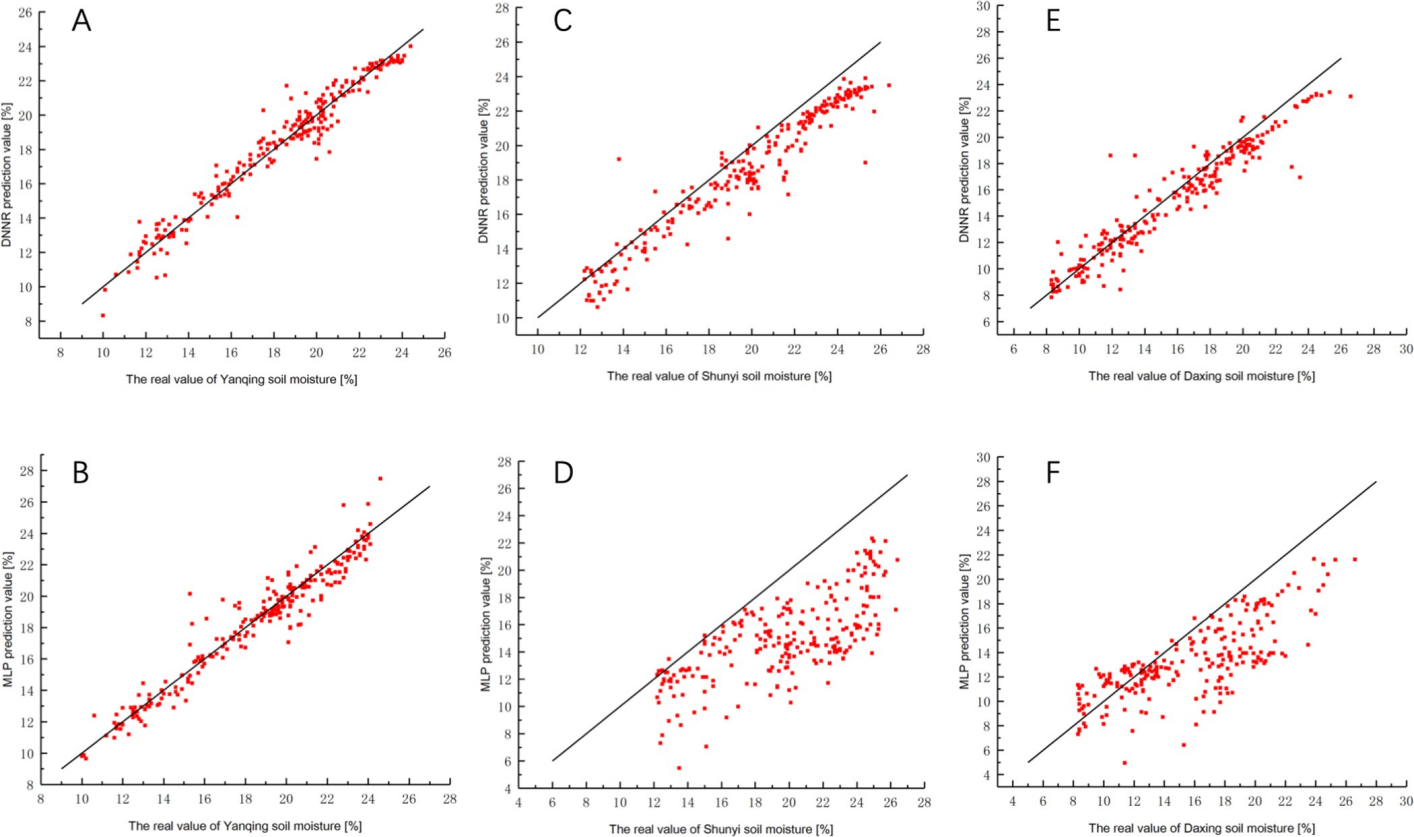
The fitting between the predictions and real values in Yanqing, Shunyi and Daxing. (A) DNNR model in Yanqing area.(B) MLP model in Yanqing area. (C) DNNR model in Shunyi area.(D) MLP model in Shunyi area. (E) DNNR model in Daxing area.(F) MLP model in Daxing area.

**Fig 11:**
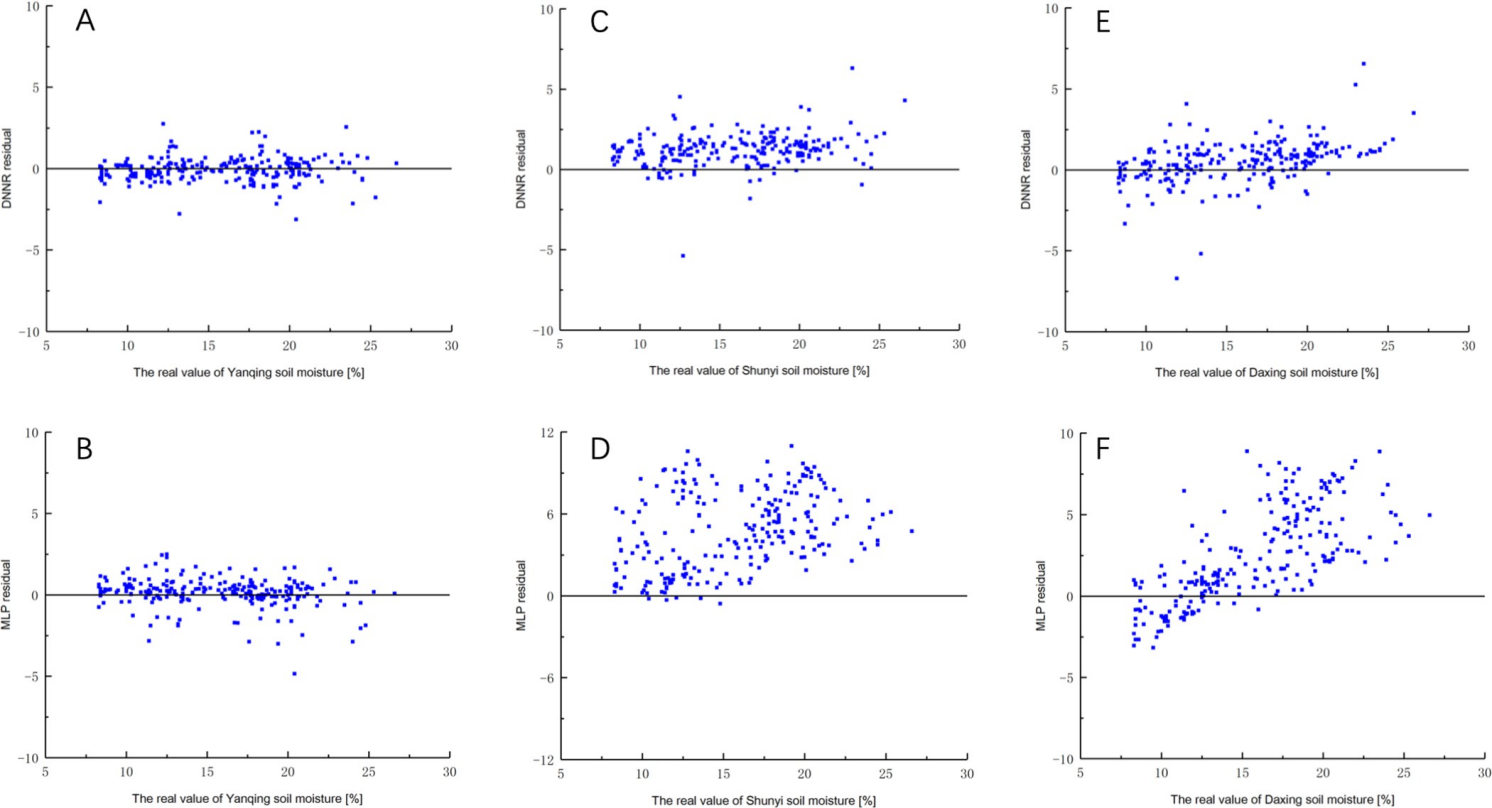
The distribution of prediction residuals in Yanqing, Shunyi and Daxing. (A) DNNR model in Yanqing area.(B) MLP model in Yanqing area. (C) DNNR model in Shunyi areae.(D) MLP model in Shunyi area. (E) DNNR model in Daxing area.(F) MLP model in Daxing area.

The MLP model error analysis results are in Table 6. All evaluation measures are weaker than the DNNR model. In addition to the great prediction results of the Yanqing area, other regional evaluation measures are difficult to accept. A further comparison of the two models is shown in Figs 10 and 11.

**Table 6:** Multi-region prediction error analysis results of MLP model.

AREA	Evaluation Measures	MLP
Yanqing	MAE	0.66
	MSE	0.93
	RMSE	0.96
	R^2^	0.97
Shunyi	MAE	4.80
	MSE	30.9
	RMSE	5.57
	R^2^	0.70
Daxing	MAE	2.92
	MSE	13.9
	RMSE	3.73
	R^2^	0.75

The comparison between DNNR and MLP predicted value-real value sets are shown in Fig 10. The value of DNNR prediction is closer than MLP to the true value. The correlation coefficient of the DNNR model for the predicted value-real value of the Yanqing area, Shunyi area and Daxing area is 0.98, 0.97 and 0.96 and higher than the MLP model is 1.03%, 38.6% and 28.0% respectively.

The comparison between DNNR and MLP predicted residual-predicted value sets are shown in Fig 11. The most of residual fluctuation range of MLP is within [-2,+2], and the relative error of prediction is 0.27%. The most of residual fluctuation range of DNNR is within [-2,+2], and the relative error is 0.11%. In the comparison of local data prediction, the performance advantages of the DNNR model are not particularly prominent. But in other regions, the soil moisture in Daxing and Shunyi areas of Beijing was predicted and compared to further research the generalization capability of the DNNR model and MLP model in soil moisture prediction application. The most of residual fluctuation range of MLP in Shunyi area and Daxing area is within [0,12] and [–3,10]. The most of residual fluctuation range of DNNR in Shunyi area and Daxing area is within [0,4] and [–2,4]. The DNN model residuals fluctuate around the zero point in a small range with only a few outliers. However the residual of the MLP model is difficult to control. The MAE of DNNR in Shunyi area and Daxing area decreased by 72.29% and 64.73% compared with MLP, the MSE of DNNR in Shunyi area and Daxing area decreased by 91.67% and 85.83% compared with MLP, the RMSE of DNNR in Shunyi area and Daxing area decreased by 71.10% and 62.47% compared with MLP. The above experiments show that under the same training set and test set conditions, the DNNR model displays better prediction accuracy than the commonly used three-layer MLP network.

In summary, this paper uses the meteorological data and initial soil moisture data of Yanqing in Beijing to construct a DNNR model to predict soil moisture, analyze the correlation between various meteorological parameters, soil water content, and the characteristics of the moisture data. Based on the analysis results, the training set and test set are constructed. Then the training model obtains the ideal result by predicting the depth of the Yanqing 20 cm depth soil moisture and is then used to predict other areas. The prediction is acceptable and meaningful. Various comparison tests prove that the DNNR model has good generalization ability and fitting accuracy. However, this experiment still needs to proceed further: (1) it needs to be further applied to more areas to verify the effectiveness of the model in predicting soil water content under different climatic conditions; (2) using mixed data to construct data sets and training models, such as fusing meteorological data and remote sensing data to analyze model feasibility; (3) increase the control experiment by changing the input features, and further analyze the impact of different meteorological characteristics on the accuracy of soil moisture prediction.

## 6. Conclusions

1. Soil moisture data is a non-stationary time series, which presents a periodic variation regular pattern involving large fluctuations. It is known from correlation analysis that each parameter characteristic has a correlation with the moisture parameter, which affects the predicted value, and that the initial soil moisture feature has the greatest weight. Humidity and temperature are second. Although the rainfall variable directly affects the soil water content, its distribution is highly random and noisy, leading to a low weight factor that cannot be used as the only fitting parameter. Therefore, the seven input variables discussed in this paper were selected as the inputs of the prediction model.
2. The deep learning model is used to predict the soil moisture at a depth of 20 cm in the Yanqing area. It was proven by experiments that too many layers of the model can lead to too excessive training time and overfitting, the latter which affects training accuracy and generality. Finally, a two-layer hidden layer was considered most suitable for our model’s structure. The first layer is responsible for learning the input features, and the second layer is responsible for polynomial fitting of the learned features, and too many nodes will cause overfitting and reduce the prediction accuracy and generalization capability. Ultimately, after ten repetitions of training, the model structure was determined to be 7-100-50-1 and the DNNR model can ensure that the overall prediction error in the Yanqing area is controlled at ±1.
3. At the same time, the DNNR model also can predict the moisture trends of other regions (Shunyi and Daxing), and has ability to keep prediction error near the zero point. All evaluation indicators are better than MLP model. The above results indicate that the DNNR model has excellent generalization capability and scalability. It is feasible to apply soil moisture prediction and provide technical support for irrigation strategies and drought control using this model.
